# Probing intergranular mixed transgranular stress corrosion cracking under the high constant load

**DOI:** 10.1038/s41598-022-16390-1

**Published:** 2022-07-20

**Authors:** Longkui Zhu

**Affiliations:** Beijing Institute of Structure and Environment Engineering, China Academy of Launch Vehicle Technology, China Aerospace Science and Technology Corporation, Beijing, 100076 China

**Keywords:** Metals and alloys, Surfaces, interfaces and thin films

## Abstract

Stress corrosion cracking (SCC) of non-sensitized austenitic stainless steel during immersion of MgCl_2_ solutions was investigated by X-ray computed tomography and scanning electron microscopy. SCC cracks propagated transgranularly at the initial stage, and switched to the IGSCC mode under the high constant load. There was no ductile dimple present on the SCC fractography, but numerous cleavage facets, slip bands and pits emerged. The cohesive zone appeared ahead of the crack tip. It is indicated that the high-load SCC also coincides with the cleavage dissolution mechanism, predominantly originating from the corrosive environment particle assisted cleavage and the obstacle induced dislocation pinning.

Brittle rupture of ductile metals, for instance stress corrosion cracking (SCC) of austenitic stainless steel in chloride solutions, usually advances on grain boundaries or crystal planes. Previous experimental results have indicated that the transgranular (TG) SCC fractography is generally river-like and crystallographic in type 316L stainless steel at the elastic stress levels^1–3^, and the TGSCC can also take place in mode I, II and III loaded austenitic stainless steel^4–6^. The two-surface trace analysis has demonstrated that the non-slipping TGSCC advances along cleavage planes {1 0 0} rather than slip planes {1 1 1}^2,3^. The earlier etch-pitting and stereographic observations show that the TGSCC predominantly grew on crystal planes {1 0 0}, and some of the cracking facets consisted of the alternating crystal planes {1 1 1}^7^. Magnin and Chateau et al.^8,9^ further analyzed the microscopic fractography of the type 316L single crystals, and proposed that the TGSCC cracks advanced along crystal planes {1 0 0} and {1 1 1} in the slow strain rate tensile tests. However, the ductile fracture originating from the alternation of crystal planes {1 1 1} in ligaments was simultaneously observed in the same tests^8^. Thus, it is difficult to distinguish between the ductile fracture and the brittle cleavage. To sum up, the non-slipping TGSCC of the type 316L austenitic stainless steel exhibits the same crystallographic features even using the diverse characterizations and analysis techniques, whereas no common understanding of the TGSCC with dislocation slipping has been arrived at until now.

Apart from the TGSCC, the intergranular (IG) SCC is probable to occur in the austenitic stainless steel as well. In particular, since the Cr-depleted grain boundaries in the sensitized austenitic stainless steel heated at 415–850 °C act as anodes and dissolve preferentially^10^, the localized stress concentrates in the vicinity of the dissolved grain boundaries, and the brittle cleavage is apt to occur on the grain boundary facets at relatively low applied stress levels. Alyousif and Nishimura^11,12^ have carried out some constant-load SCC experiments in the sensitized austenitic stainless steel immersed into the boiling MgCl_2_ solutions. For the type 304 sensitized austenitic stainless steel, the average IGSCC stress level of 300 MPa was higher than the average TGSCC stress level of 200 MPa^11^. At the average stress level of 300 MPa, the IG- and TGSCC of the type 316 sensitized austenitic stainless steel took place in the 133 °C and 145 °C boiling MgCl_2_ solutions, respectively^12^. Subsequently, Sarvesh Pal and Singh Raman^13^ also discovered that the IGSCC initiated and propagated with a few accompanying TGSCC phenomena in the type 304 sensitized austenitic stainless steel. These experimental results indicate that the sensitized austenitic stainless steel is susceptible to both the IG- and TGSCC. More interestingly, for the type 304 solution-annealed austenitic stainless steel, the SCC cracks nucleated transgranularly at the initial stage, and switched to the IGSCC mode with increasing the crack-tip stress under the constant load^13^. It is indicated that the IGSCC of non-sensitized austenitic stainless steel becomes another probably-existing phenomenon, and the corresponding mechanism is necessarily elucidated at length.

Furthermore, whether the IGSCC or the TGSCC occurs obviously depends on the cohesive forces between the SCC facets in the cohesive zones. Ordinarily, two parts of the cohesive forces are summarized as the surface energy and the plastic deformation work. The experimental phenomena have manifested that the SCC can initiate and propagate on cleavage planes (1 0 0) without dislocation slipping at the elastic stress levels^1–3,14^. The atomic-scale simulations have shown that synergistic adsorption of H and Cl atoms in the octahedral interstices minimizes the surface energy of the cleavage planes (0 0 1) owing to a 73% reduction, while the surface energy only decreases by 28% under the condition of hydrogen adsorption in the face-centered cubic lattices of the FeCrNi alloy roughly equivalent to the type 316L austenitic stainless steel^3^. For accurate elucidation and prediction of the damage, the cleavage dissolution model has been put forward, proposing that the TGSCC rupture is attributable to the brittle cleavage on the low surface energy planes, and the dislocation pinning in the presence of corrosion environment particles (CEPs) such as hydrogen atoms and electronegative ions^3^. Similarly, for Al (1 1 1) and Fe (1 1 0), the hydrogen or oxygen atoms have also been manifested to result in the tremendous reduction of the metal cohesion^15–17^, and the probable transition from the atomic bonding forces to the van der Waals forces during decohesion and cleavage under the condition of no hydrogen^16^. The calculations have further confirmed that the grain boundary in the nickel bicrystal containing hydrogen is able to cleave without dislocation motion or amorphization, but the intergranular cracking is coupled with dislocation emission in the no-hydrogen condition^18^. In brief, the CEPs are favorable to the brittle cleavage of the ductile metals with no accompanying occurrence of the dislocation slipping. On the other hand, it is discernible that the environment assisted fracture inevitably consists of the brittle cleavage and the ductile shear, when the applied shear stress exceeds the critical resolved shear stress to activate a slip system^2,4^. Nevertheless, the effect of ductile shear on the high-load SCC remains disputed, and needs to be further clarified in theory.

As mentioned above, abundant efforts have been devoted to the damage of the austenitic stainless steel in chloride-contained environment, but the microscopic mechanisms on the high-load SCC are still of unawareness. This article first aims to characterize two- and three-dimensional crack morphologies and analyze microscopic fractography of the high-load SCC in the type 316L non-sensitized austenitic stainless steel. Next, the relations between the brittle cleavage and the ductile shear are elucidated in detail. The issues concerning the consistence and the distinction between the IGSCC and the TGSCC are also discussed at the final.

## Results

### SCC cracks and fracture surfaces

**Figure 1:**
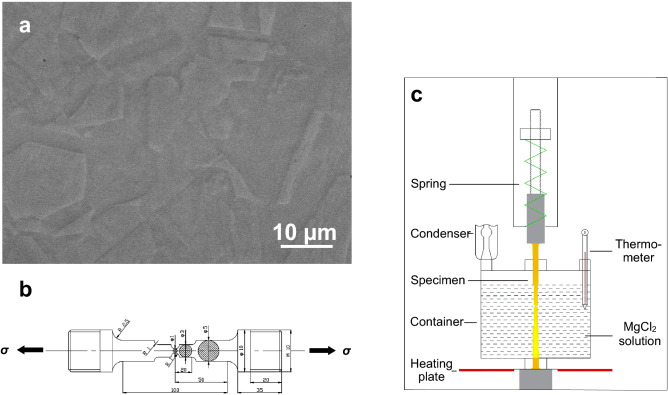
Materials and the setup. **a** Metallographic morphology of type 316L austenitic stainless steel. **b** Schematic diagram of the cylinder specimen subjected to a constant nominal stress of *σ*_nom_ = 120 MPa ± 10 MPa in the narrowest section with the approximately 1 mm diameter. **c** Schematic diagram of the spring-loaded SCC setup.

**Figure 2:**
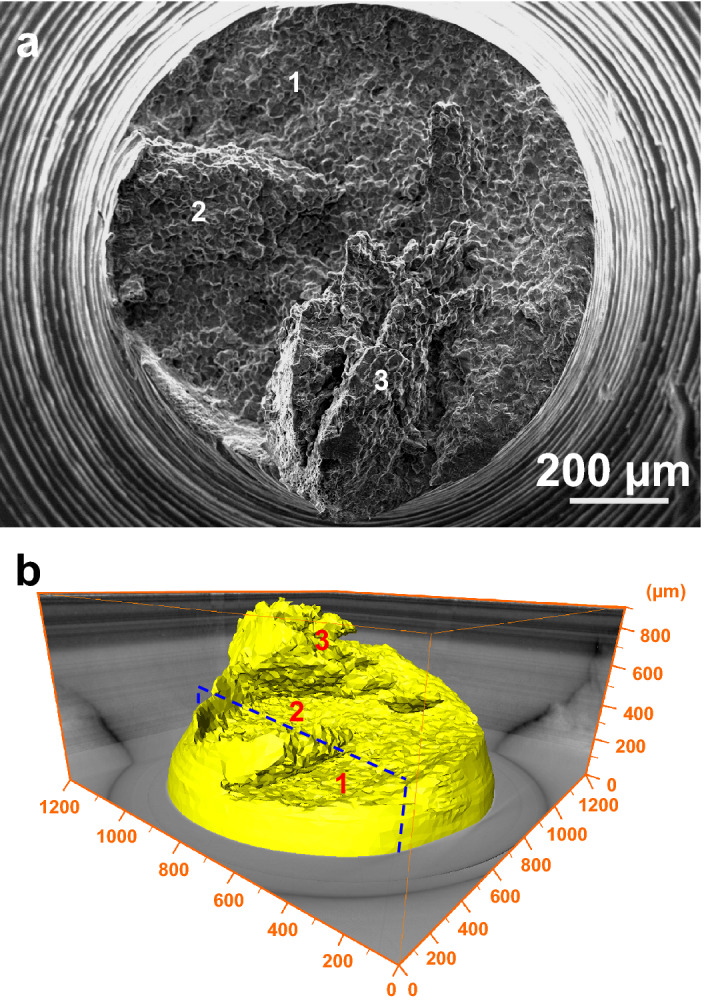
Two- and three-dimensional fracture surface morphologies of the upper half part of an initially-fractured specimen subjected to the chloride-solution immersion of about 10 h. **a** Two-dimensional SEM morphology. **b** Three-dimensional XCT morphology consisting of three fracture surfaces marked by ‘1–3’ and a tomographic slice labeled by a dotted rectangle.

**Figure 3:**
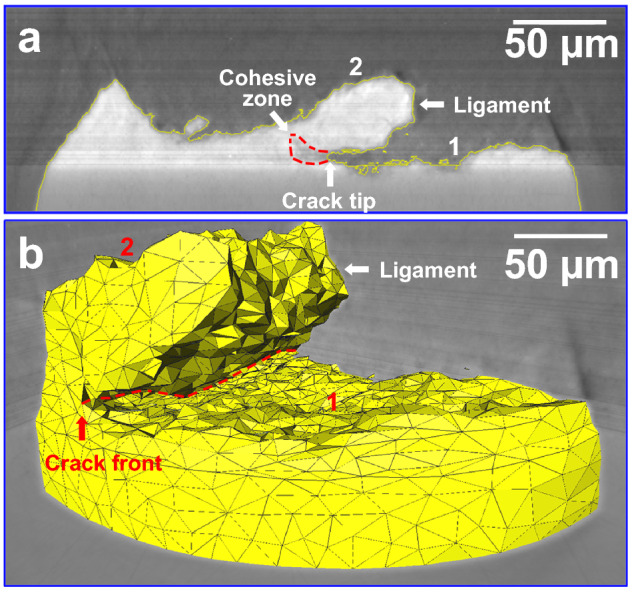
Two- and three-dimensional SCC crack morphologies characterized by the XCT. **a** The two-dimensional section morphology of the tomographic slice labeled by the dotted-rectangle in Fig. 2b. **b** The three-dimensional local morphology ranging from the dotted-rectangle tomographic slice to the specimen surface. It is indicated that there was a cohesive zone ahead of the SCC crack tip.

As shown in Fig. 1, the type 316L non-sensitized austenitic stainless steel subjected to *σ*_nom_ = 120 MPa ± 10 MPa was immersed into the boiling 45 wt.% ± 1 wt.% MgCl_2_ solution. The tested specimens fractured after the chloride solution immersion of 2–10 h. As seen in Fig. 2a, for the upper half part of the specimen with the fracture time of about 10 h, the diameter of the macroscopic fractography was approximately 1 mm, almost equal to the narrowest section diameter of the original specimen. Simultaneously, the necking deformation was slighter on the side surface adjacent to the ‘1’-marked fracture surface than near the ‘2’- and ‘3’-marked fracture surfaces, shown in Fig. 2b. Both of the two- and three-dimensional SCC morphologies indicates that there were three fracture surfaces marked by ‘1–3’ on the macroscopic fractography. Furthermore, the two-dimensional section perpendicular to the ‘1’- and ‘2’-marked fracture surfaces was drawn from the tomographic slices, shown in Fig. 3a. There was a ligament between the ‘1’- and ‘2’-marked fracture surfaces. Especially, a cohesive zone, identically named by the elastic stress concentration zone^3^ or the plastic zone^9^, emerged ahead of the SCC crack tip, which might also contain the CEPs such as chloride ions and hydrogen atoms in the light of the difference of the X-ray adsorption coefficients. Additionally, from the three-dimensional viewpoint, the corresponding SCC crack front was curved instead of being linear, shown in Fig. 3b.

### IGSCC and TGSCC fractography

**Figure 4:**
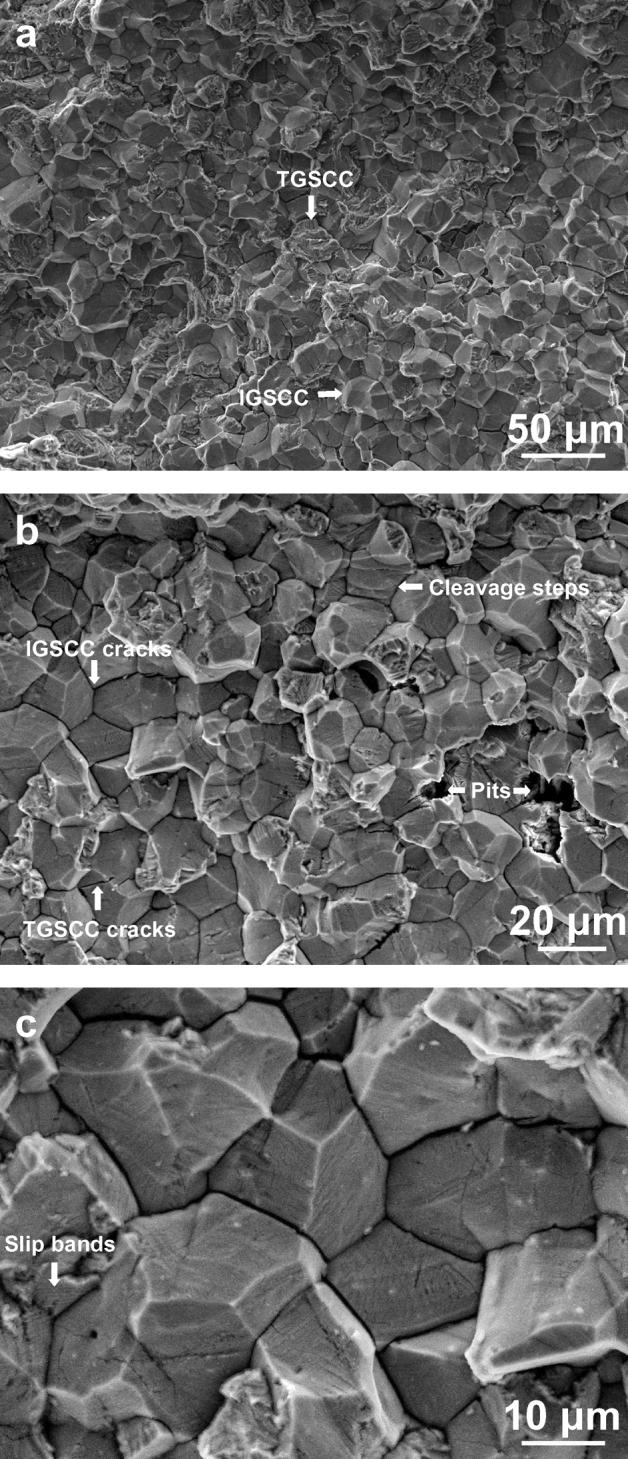
Microscopic fractography of the upper half part of an initially-fractured specimen rinsing in acetone and cleaning in deionized water. **a** The IG- mixed TGSCC. **b** The SCC cracks, the cleavage facets and the pits. **c** The slip bands during the IGSCC at the high stress level. It is apparent that no ductile dimples presented on the fracture surfaces.

**Figure 5:**
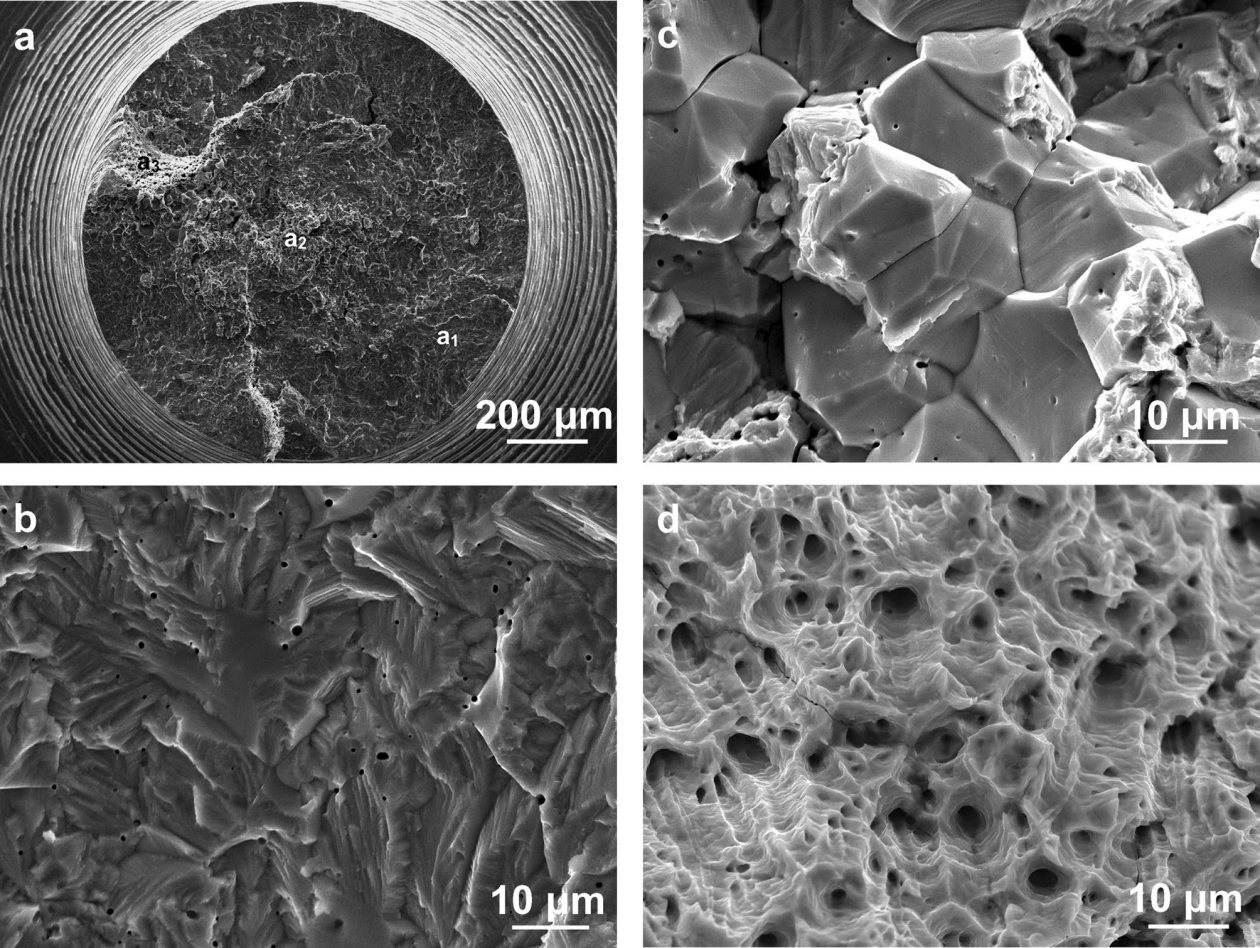
Macro- and microscopic fractography of another initially-fractured specimen rinsing in acetone and cleaning in deionized water. **a** Macroscopic fractography. **b** Microscopic TGSCC fractography in the zone ‘a_1_’. **c** Microscopic IG- mixed TGSCC fractography in the zone ‘a_2_’. **d** Ductile-dimple fractography in the zone ‘a_3_’, where the predominant TGSCC initiated in the zone ‘a_1_’, subsequently propagated to the accompaniment of the IG- mixed TGSCC mode in the zone ‘a_2_’, and ultimately the specimen fractured in the zone ‘a_3_’ after the chloride-solution immersion of about 7 h.

**Figure 6:**
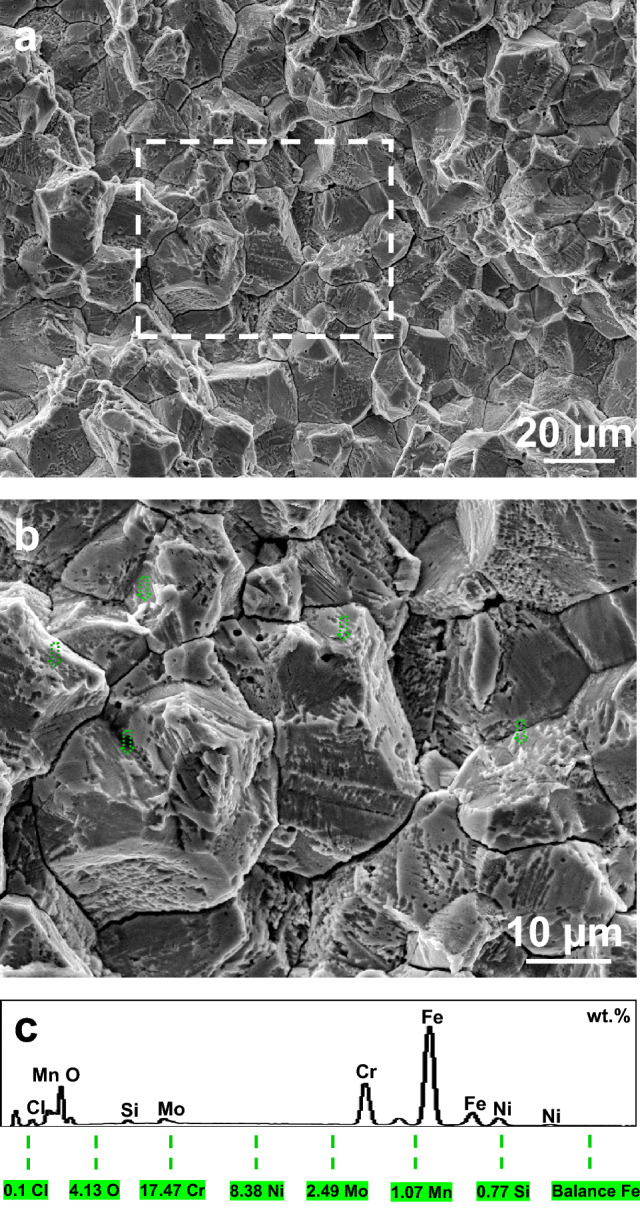
Microscopic fractography and corrosion product composition of the initially-fractured lower half part continuously corroded for about 10 h. **a** The corroded fracture surface. **b** The stochastically-distributed arrow-labelled corrosion product in the area marked by the dotted rectangle in a. **c** The corrosion product composition analyzed by the EDS.

The microscopic fractography of the fractured specimens was exactly examined by the SEM. Akin to the specimen shown in Fig. 4a, all of the fractured specimens were susceptible to the IGSCC mixed with the TGSCC, and simultaneously there was no ductile dimple present in the IGSCC and TGSCC zones. It is also found that numerous IGSCC cracks, TGSCC cracks, cleavage facets and localized pits emerging on the microscopic fractography, shown in Fig. 4b. To clearly elucidate the high-load SCC mechanisms, the higher-resolution fractographic morphology was further detected in Fig. 4c. Most of the grain boundary facets were very flat in spite of some micron-scale-interval stripes existing on the facets. Meanwhile, the evident slip bands testified to occurrence of the ductile shear during the high-load fracture process. These unique phenomena reappeared in another fractured specimen after the chloride-solution immersion of about 7 h, shown in Fig. 5. The radial stripes show that the TGSCC primarily consisting of cleavage steps first initiated in the zone ‘a_1_’ on the macroscopic fractography. Next, the IGSCC mixed with the TGSCC occurred to the accompaniment of flat facets in the zone ‘a_2_’ with the increase of the crack-tip stress under the constant load. Ultimately, the ductile dimples were formed in the zone ‘a_3_’ and the specimen fractured instantly. In addition, the corrosion product containing the oxygen and chlorine elements in Fig. 6 was stochastically distributed on the crystal planes or the grain boundary facets of the lower half part continuously corroded for about 10 h after fractured.

## Discussion

The experimental observations as mentioned above indicate that both rupture on cleavage facets and gliding along slip planes occurred during the SCC processes at the high stress levels exceeding the critical resolved shear stress. The initiation and propagation of SCC cracks becomes a somewhat complex phenomenon on invoking a combination of the brittle cleavage and the ductile shear. On one hand, the metal-crystal SCC is a type of brittle fracture mode in essence^19,20^. The absolute brittle phenomenon is the non-slipping SCC advance at the elastic stress levels^1–4,14^, and the CEP assisted van der Waals phase transition during decohesion and cleavage^16^. In the experiments, the microscopic IGSCC fractography, primarily consisting of flat cleavage facets but no ductile dimples, actually provides the validation of the brittle cleavage during the high-load SCC. What’s more, considering localized pitting, the cleavage dissolution model is suitable for the predominant processes of the high-load SCC, which proposes that the initiation and propagation of SCC cracks emanate from the brittle rupture on the low-surface-energy cleavage facets in the cohesive zones, and the anodic dissolution along the SCC crack fronts^3^. Wherein, the intrinsic dislocations are pinned by the CEPs, leading to increase of the critical resolved shear stress and embrittlement of the cohesive zones^3^. Distinct from the cleavage dissolution model, however, most of the previous SCC mechanisms, such as the slip dissolution model and the unified model of environment-assisted cracking, neglect the essential cleavage at the high stress levels^21–23^.

**Figure 7:**
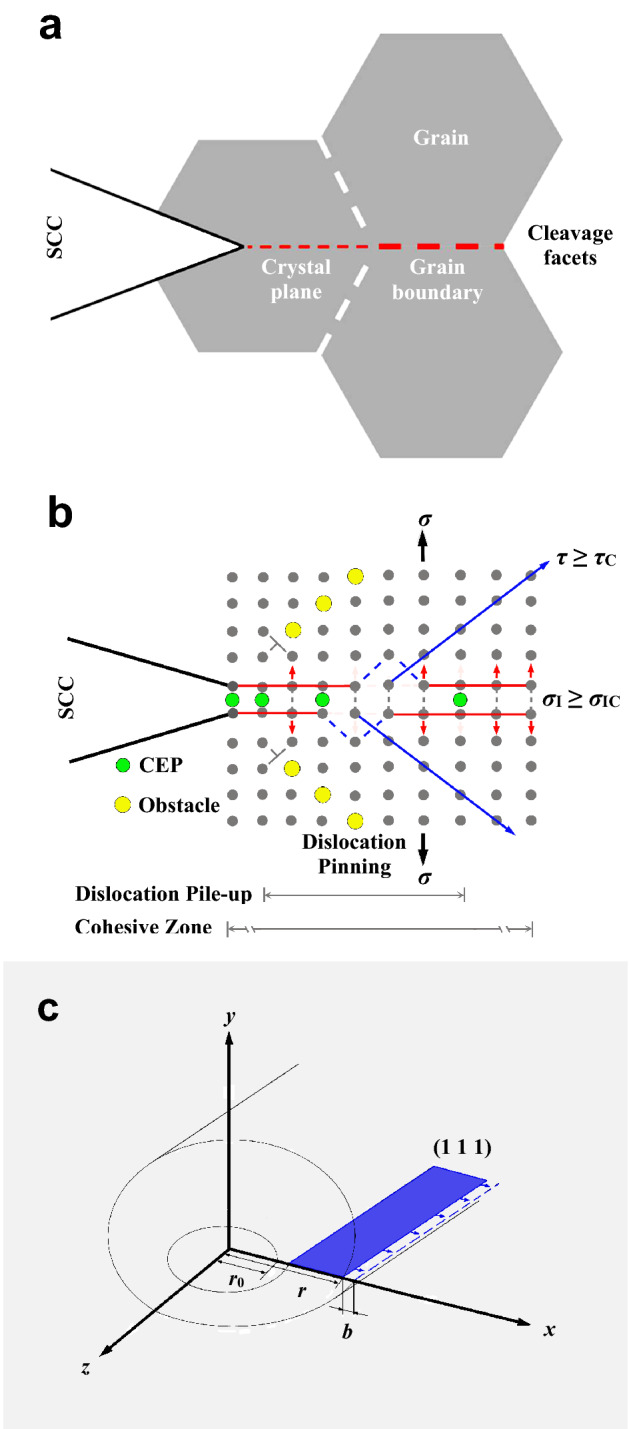
The mechanism model of an IG- mixed TGSCC process. **a** A SCC crack on two cleavage facets consisting of a crystal plane and a grain boundary. **b** Synergistic effects of the CEP assisted cleavage on *σ*_I_ ≥ *σ*_IC_ and the ductile shear on *τ* ≥ *τ*_C_ in the elastic–plastic cohesive zone, in which some dislocations are pinned by the obstacles or pile up ahead of the SCC crack tip. **c** A slip plane (1 1 1) gliding by a Burgers vector, *b*, in a canister-like elastic strain field induced by an edge dislocation. Wherein, the CEPs are composed of the hydrogen atoms and the electronegative ions such as the chloride ions for the chloride-solution SCC of the austenitic stainless steel, while the obstacles contains the grain boundaries, the CEPs, the precipitates, the Cottrell or Snoek atmospheres and the L-C dislocation locks.

On the other hand, the cleavage dissolution model doesn’t rule out the ductile shear either when the applied shear stress exceeds the critical resolved shear stress. The relations between the brittle cleavage and the dislocation slipping become fascinating due to no ductile dimples formed on the high-load IGSCC and TGSCC fractography. Overall, the predominant high-load SCC mechanism still remains the CEP assisted cleavage on crystal planes or grain boundaries under the normal stress, and simultaneously some of the unpinned or pinned dislocations are activated to glide along the slip planes under the high shear stress, schematically illustrated in Fig. 7a and b. Wherein, a few gliding dislocations induce the formation of the slip bands on the specimen surfaces^2,4^ or the facture surfaces, and more movable dislocations pile up around the obstacles such as the grain boundaries, the CEPs, the precipitates, the Cottrell or Snoek atmospheres and the L-C dislocation locks. These dislocation pile-ups further impinge on the dislocation motion and instead increase the localized plastic deformation work. Subsequently, the CEP-assisted low-surface-energy facets subjected to the concentrated normal stress occurs to cleave on the crack fronts or at the dislocation pile-up sites according to the stroh’s mechanism. The experimental observations have revealed that the microcracks either initiate in the dislocation-free zones between the crack fronts and the dislocation pile-up sites^24,25^. By contrast, if the metal crystals are barely loaded in the non-corrosive environment, the microcaves alone nucleate on the crack fronts or in the dislocation-free zones, which eventually forms the ductile dimples on the fractography^26^. It is concluded that the CEP-assisted cleavage rather than the dislocation slipping is properly the essential distinction between the SCC in the corrosive environment and the ductile fracture in the non-corrosive environment. For the high-load SCC, synergistic effects of the CEP assisted cleavage and the obstacle induced dislocation pinning actually provide systematic insight into the brittle rupture and the fractography without ductile dimples. Besides, an emphasis needs to be laid on the CEPs composed of the hydrogen atoms and the electronegative ions such as the chloride ions for the chloride-solution SCC of the austenitic stainless steel^3^.

For convenience, a high-load SCC process can be roughly divided into three main parts: the brittle cleavage, the anodic dissolution and the ductile shear. Namely, the total crack propagation rate, *v*, is:

1 $$v = \alpha_{1} v_{{\text{c}}} + \alpha_{2} v_{{\text{a}}} + \alpha_{3} v_{{\text{s}}}$$

where *v*_c_, *v*_a_ and *v*_s_ denote the crack propagation rates separately associated with the brittle cleavage, the anodic dissolution and the ductile shearing, *α*_1_, *α*_2_ and *α*_3_ denote the corresponding percentages of each part, which can also be defined as the coefficients of *v*_c_, *v*_a_ and *v*_s_, respectively. The corresponding values ranging from 0 to 1 actually vary with the applied stress levels. If the applied stress is kept constant, each parameter also amounts to a fixed value and the sum of them is 1. For instance, *α*_3_ = 0 under the elastic loads, and *α*_3_ is less than 1 at the high stress levels. Obviously, *α*_3_ ≠ 1, otherwise the fracture mode is not the SCC. Furthermore, the ductile shear also contains two types including the slipping and the twinning. Both of them are essentially caused by the gliding of the slip planes {1 1 1} in the face centered cubic lattices. The critical resolved shear stress, *τ*_C_, of the partial dislocation induced twinning, is fairly larger than that of the perfect dislocation induced slipping. For the localized slipping, the theoretical critical resolved shear stress, *τ*_C_, is:

2 $$\tau_{{\text{C}}} = \frac{Ga}{{2{\uppi }d}}$$

where *G* is the shear modular, *a* is the atomic spacing on the slip planes {1 1 1}, and *d* is the distance between the slip planes {1 1 1}, *d* = (1^2^ + 1^2^ + 1^2^)^−1/2^*a* = 3^−1/2^*a*. Thus, the theoretical critical resolved shear stress amounts to 0.28*G*. In practice, *τ*_C_ of the macroscopic specimens is about 10^−4^ ~ 10^−3^ times as large as their shear modular owing to various intrinsic defects distributed within the crystals^2,27^. When the applied shear stress, *τ*, approaches *τ*_C_, the slip systems can be activated and the dislocations occur to motion. As shown in Fig. 7c, assuming that an edge dislocation glides along the *x*-axis direction at a distance of *x* during a period, *t*, *τ* is:

3 $$\tau = \frac{Gb\beta }{{2{\uppi }(1 - \nu )}}\frac{1}{x}$$

where *b* is the Burgers vector, *β* is the coefficient of the Burgers vector related to *x*, 0 ≤ *β* ≤ 1, *ν* is the Poisson’s ratio. As shown in Fig. 7c, if the elastic strain field around the edge dislocation approximates to a canister with the internal diameter of *r*_0_ and the external diameter of *r*, the surrounding stain energy, *W*_surrounding_, can be given by:

4 $$W_{{{\text{surrounding}}}} = \int_{0}^{1} {\int_{{r_{0} }}^{r} {\frac{{Gb^{2} \beta }}{{2{\uppi }(1 - \nu )}}} } \frac{{{\text{d}}\beta {\text{d}}x}}{x} = \frac{{Gb^{2} }}{{4{\uppi }(1 - \nu )}}\ln \frac{r}{{r_{0} }}$$

On calculating the total strain energy including the surrounding strain energy and the core strain energy (≤ *r*_0_), *r*_0_ can be approximated to:

5 $$r_{0} = \lambda b$$

where *λ* ≈ 0.25 for the metals. Submitting Eq. (5) to Eq. (4) yields the total strain energy, *W*_total_:

6 $$W_{{{\text{total}}}} = \frac{{Gb^{2} }}{{4{\uppi }(1 - \nu )}}\ln \frac{r}{\lambda b}$$

Further, if a crack initiates accompanied by the dislocation motion, the plastic deformation work, *γ*_P_, is equivalent to *W*_total_. That is:

7 $$\gamma_{{\text{P}}} = W_{{{\text{total}}}} = \frac{{Gb^{2} }}{{4{\uppi }(1 - \nu )}}\ln \frac{r}{\lambda b}$$

According to the Orowan’s and Irwin’s theory, the crack growth resistance, *R*, is:

8 $$R = 2\gamma_{{\text{S}}} + \gamma_{{\text{P}}} = 2\gamma_{{\text{S}}} + \frac{{Gb^{2} }}{{4{\uppi }(1 - \nu )}}\ln \frac{r}{\lambda b}$$

where *γ*_S_ is the surface energy of the fresh cracked facet. As for the mixed dislocation, assuming that *b* is the Burgers vector and *φ* is the angle between the Burgers vector and the dislocation line, the Burgers vectors of the edge dislocation part and the screw dislocation part are *b*sin*φ* and *b*cos*φ*, respectively. Under the circumstance, *R* can be transformed to:

9 $$R = 2\gamma_{{\text{S}}} + \frac{{Gb^{2} }}{{4{\uppi }}}\left( {\frac{{\sin^{2} \phi }}{1 - \nu } + \cos^{2} \phi } \right)\ln \frac{r}{\lambda b}$$

Since *γ*_P_ is significantly greater than *γ*_S_, the obstacle induced dislocation pinning has a tremendous impact on decreasing the stress thresholds of the SCC without or only locally coupled with the dislocation slipping. Therefore, once the intrinsic dislocations or some of them are pinned inside the cohesive zones, the cleavage microcracks would like to initiate on the CEP-assisted low-surface-energy facets under the applied stress far below the fracture strength. Moreover, the mixed dislocation accompanying crack propagation rate, *v*_s_, in the light of Eq. (3) can be given by:

10 $$v_{{\text{s}}} = \frac{Gb}{{2{\uppi }\tau }}(\frac{{\sin^{2} \phi }}{1 - \nu } + \cos^{2} \phi )\frac{{{\text{d}}\beta }}{{{\text{d}}t}}$$

As a consequence, submitting Eq. (10) into Eq. (1), the total crack propagation rate during the high-load SCC process can be written as:

11 $$v = \alpha_{1} v_{{\text{c}}} + \alpha_{2} v_{{\text{a}}} + \alpha_{3} \frac{Gb}{{2{\uppi }\tau }}\left( {\frac{{\sin^{2} \phi }}{1 - \nu } + \cos^{2} \phi } \right)\frac{{{\text{d}}\beta }}{{{\text{d}}t}}$$

As to *v*_c_ and *v*_a_, both of the two parameters can be substituted with the corresponding functions described in the previously-reported literature^3^.

Additionally, another meaningful issue is how to essentially distinguish between the IGSCC and the TGSCC. The obtained experimental results also indicate that both the IG- and TGSCC at the high stress levels are attributable to the brittle cleavage, the anodic dissolution as well as the ductile shear. On the basis of the cleavage dissolution model, it is summarized that the consistent features of the IG- and TGSCC are the CEP assisted cleavage and the obstacle induced dislocation pinning^3^. Apart from their consistence, the following is the distinction between the IGSCC and the TGSCC. From the perspective of the obstacle induced dislocation pinning, the obstacles for the elastic-load TGSCC mainly consist of the CEPs, while those for the plastic-load TGSCC are also proposed to include the dislocation pile-up sites except the CEPs. Moreover, the grain boundaries are regarded as the other obstacles for the IGSCC owing to the shield of dislocation motion. For the type 316L non-sensitized austenitic stainless steel, the grain boundaries usually contain various microscopic defects such as dislocations, vacancies, precipitates and so on^28,29^. The dislocations act as the mass transport paths^13,30^, leading to the faster diffusion of the CEPs on the grain boundaries than inside the grains. It is implied that the CEP adsorption on the grain boundary facets tends to be saturated in the shorter period. Although the surface energy is originally larger on the grain boundary facets with the complicated crystallography than that of the low-surface-energy crystal planes within the grains, the CEP assisted surface energy of the grain boundary facets might conversely lower that of the crystal planes during the same time. Simultaneously, the grain boundaries with the dislocation pile-ups do not only block further motion of the dislocations and decrease the critical cracking stress, but also appropriately promote the localized normal stress concentration. Like this, the IGSCC prior to the TGSCC is apt to nucleate in the type 316L non-sensitized austenitic stainless steel subjected to the high applied stress.

## Conclusions

Numerous flat grain boundary facets and cleavage steps on the fractography show that the IG- mixed TGSCC occurred in type 316L non-sensitized austenitic stainless steel subjected to the high constant load during the chloride-solution immersion of 2–10 h. The predominant mode transferred from the TGSCC to the IGSCC with increasing the crack-tip stress, and the ductile dimples were ultimately formed in the instant fracture zone. Some slip bands and localized pits were coupled with the high-load SCC. It is also found that there was the cohesive zone ahead of the crack tip. The stochastically-distributed corrosion product contained the oxygen and chlorine elements. These experimental observations indicate that the high-load SCC initiates and propagates in accordance with the cleavage dissolution model, proposing that both the IG- and TGSCC consistently originate from the CEP assisted cleavage and the obstacle induced dislocation pinning at the scales of the microns.

## Materials and methods

The type 316L non-sensitized austenitic stainless steel were used in this study with the following chemical composition: C-0.007 wt.%, Cr-17.40 wt.%, Ni-10.47 wt.%, Mo-2.51 wt.%, Mn-1.03 wt.%, Si-0.45 wt.%, P-0.008 wt.%, S-0.0056 wt.%, Fe-balance. Few precipitates were distributed on the boundaries of the grains with the average size of about 18 μm, shown in Fig. 1a. The cylinder specimens with the 1-mm-diameter narrowest section were prepared by the following method: wire-electrode cutting; rinsing in acetone; cleaning in deionized water; drying in hot air and storing in a desiccated chamber. The schematic diagram of the cylinder specimen is shown in Fig. 1b. According to the ASTM G36-94 (2018), the specimen subjected to the nominal stress of *σ*_nom_ = 120 MPa ± 10 MPa applied by a spring-type constant load setup (in Fig. 1), was immersed into a boiling 45 wt.% ± 1 wt.% MgCl_2_ solution in an open circuit condition. Wherein, the condenser was exploited to keep the solution density constant in the glass container. During the SCC processes, the tested specimens were visually examined every about 2 h, and the fracture time approximated to the mean time of the last recorded time before fracture and the first recorded time after fracture. The SCC tests were successfully repeated for three times in the same experimental condition. For an initially-fractured vertical specimen, the upper half part was taken out as soon as possible to prevent the fractured specimen from general corrosion, which was eventually characterized by the X-ray computed tomography (XCT). The lower half part continued to be corroded for about 10 h before removed from the boiling MgCl_2_ solution. After the testing, all of the fractured specimens were ultrasonically rinsed in acetone and cleaned in deionized water. The two-dimensional SCC morphologies, together with the chemical composition of the corrosion product, were analyzed by the scanning electron microscopy (SEM) and the energy dispersive X-ray spectroscopy (EDS).

Subsequently, the synchrotron based XCT on the BL13W1 beam line at the Shanghai Synchrotron Radiation Facility was used to further visualize the three-dimensional SCC morphologies. A monochromatic X-ray beam was utilized with the energy of 50 keV, and a high-speed camera recorded transmitted intensity in a 4 s exposure/projection interval, while the sample was rotated in 0.2° increments. During each 180° rotation, 900 two-dimensional radiographs were recorded and applied to the reconstruction of slices nearly perpendicular to the tensile stress direction. Isotropic voxels with the resolution of 0.65 μm were achieved in the reconstructed slices. Next, image analysis, visualization and three-dimensional rendering were conducted using a commercial software package (Amira).

## Data Availability

All data that support the findings of this study are available from the corresponding author upon reasonable request.
